# Case Report: Totally Laparoscopic Resection of Retroperitoneal Paraganglioma Masquerading as a Duodenal Gastrointestinal Stromal Tumor

**DOI:** 10.3389/fsurg.2021.586503

**Published:** 2021-03-09

**Authors:** Zhi Zhang, Zhengbin Tu, Zhiqiang Lv, Yang Luo, Jianmao Yuan

**Affiliations:** Department of General Surgery, Suzhou Ninth People's Hospital, Affiliated Wujiang Hospital of Nantong University, Suzhou, China

**Keywords:** retroperitoneal tumor, retroperitoneal paraganglioma, laparoscopy, gastrointestinal stromal tumor, case report

## Abstract

**Introduction:** Retroperitoneal paraganglioma (RPGL) is a rare clinical tumor derived from the retroperitoneal sympathetic paraganglion tissue. Since RPGLs are locate deeply and have no specific symptoms and imaging manifestations at the early stage, which easily causes missed diagnosis or misdiagnosis. In addition, reports on totally laparoscopic resection of RPGLs are scarce due to their close proximity to large vessels, giant size, uncertain location, and unknown malignant status.

**Case Presentation:** We present here the case of totally laparoscopic resection of a 6.4 × 5.4 cm RPGL that was discovered during a workup for discomfort and upper abdominal pain in a 68-year-old female patient, mimicking a gastrointestinal stromal tumor (GIST) of the duodenum, Which was confirmed as a RPGL based on the histopathological and immunohistochemical findings.

**Conclusions:** RPGL is a rare tumor, and the transperitoneal laparoscopic approach for the RPGL is a safe, applicable method with less trauma and quick recovery, which is worth clinical popularizing and application. Moreover, the survival prognosis of RPGL patients are related to metastasis, and lifelong follow-up should be emphasized.

## Introduction

Paraganglioma is also known as ectopic pheochromocytoma, a low incidence rate of neuroendocrine tumors which located at different areas along the sympathetic and/or parasympathetic chain, however, the most common location has been the para-aortic region (1). RPGL is a rare entity, which often lacks of typical clinical symptoms related to the excessive secretion of catecholamines and unique radiologic features, making its diagnosis unclear and easy to be misdiagnosed as other neoplasms, such as duodenal GIST, retroperitoneal sarcoma, neurofibroma, pancreatic neoplasm and lymphoma, etc. (2–5). And as for surgical treatment, RPGLs are often found in close to the abdominal aorta or inferior vena cava, rendering removal difficult. In the past, these retroperitoneal tumors have been frequently managed by open surgical exploration, and minimally invasive resection is challenging, even for a senior surgeon. Report on the application of laparoscopic approach in the treatment of RPGL remains limited (6–8). We present here the case of totally laparoscopic resection of a RPGL that was discovered during a workup for discomfort and upper abdominal pain in a 68-year-old female patient, mimicking a GIST of the duodenum, combined with a laparoscopic cholecystectomy for gallstones.

## Case Presentation

A 68-year-old woman was admitted to our hospital with discomfort and upper abdominal pain that had started 2 weeks before. She had a past history of hypertension and type-2 diabetes mellitus, which were treated with Nifedipine sustained-release tablets and Metformin Hydrochloride tablets respectively. The patient did not experience nausea, vomiting and diarrhea.

**Figure 1:**
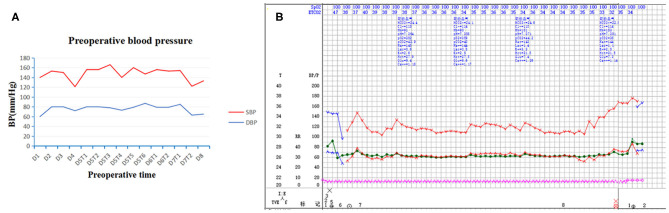
Preoperative and intraoperative blood pressure. Paroxysmal hypertension was not noticed in preoperative blood pressure **(A)**. Intraoperative blood pressure was stable throughout surgery **(B)**. SBP indicates systolic blood pressure; DBP, diastolic blood pressure; D, day; T, time.

Physical examination revealed abdominal distension, blood pressure (BP) about (140/90–160/110) mmHg, paroxysmal hypertension was not noticed (Figure 1A) and fasting blood glucose was 5.9 mmol/L. Mild tenderness in the epigastrium was noticed.

Laboratory investigations reveled a normal white blood cell count and the tumor markers such as CEA, CA199, AFP and other laboratory tests were found in normal reference ranges.

**Figure 2:**
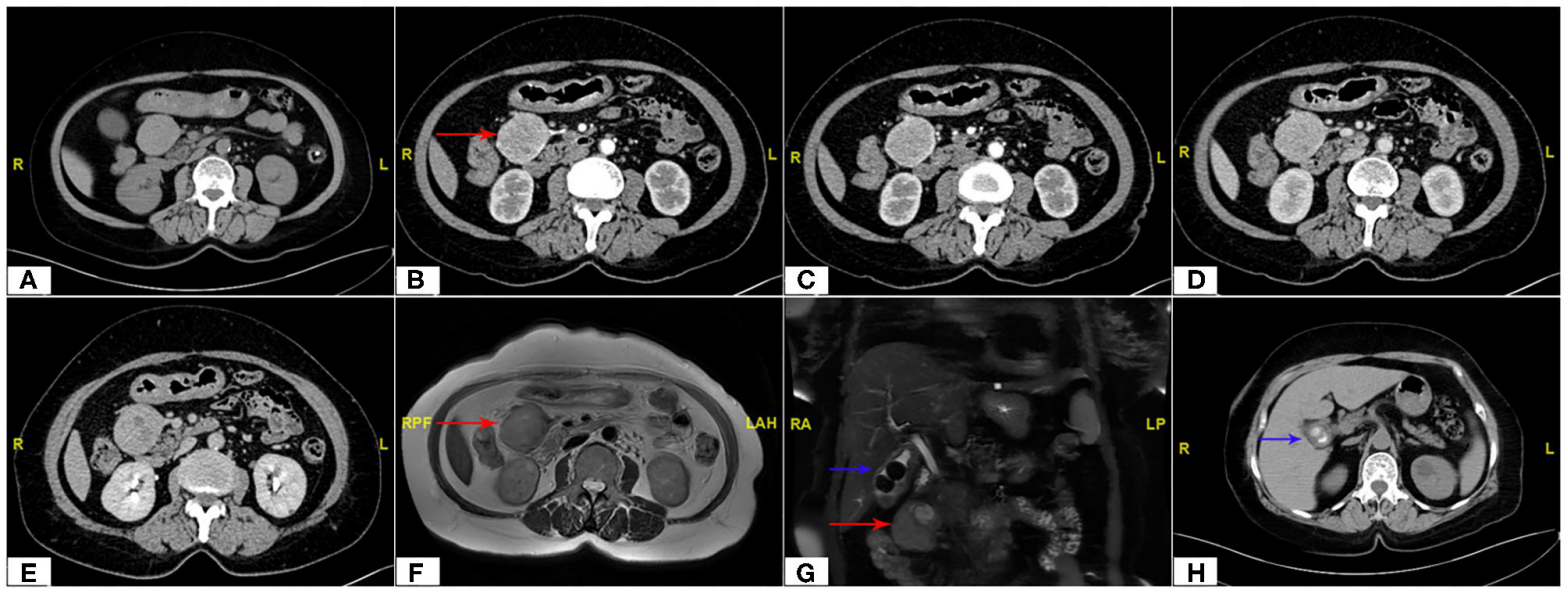
Abdomen CT scan showing the maximum cross section and the main blood supply vessels of the tumor (red arrow). **(A)** Non Contrast, **(B,C)** Arterial Phase, **(D)** Venous Phase, **(E)** Delay Phase; MRI imaging **(F,G)** of tumor (red arrow) and gallstones (blue arrow); CT scan of gallstones **(H)**.

The continuous dynamic computed tomography (CT) images in Image Database showed an isodense tumor in front of the head of pancreas, ~5.9 × 4.2 cm in size, at the same time, gallstones were found in CT imaging. The image result of magnetic resonance imaging (MRI) is the same as that of CT. Subsequent continuous dynamic enhanced CT revealed the boundary between the local area of the tumor and the duodenum was unclear and markedly inhomogeneous enhancement showed on CT imaging, which demonstrated the following feeding vessels: a branch of the superior mesenteric artery and gastroduodenal artery (Figure 2). Furthermore, the patient underwent duodenoscopy to clarify the location of the tumor relative to the duodenum and pancreas, which confirmed no obvious abnormality at duodenal papilla. Combined with enhanced CT imaging features and duodenoscopy, subserosa type of duodenal GIST was considered.

Then the patient was scheduled for laparoscopic surgery to resect the suspected subserosa type of duodenal GIST, and the procedure was converted to laparotomy if indicated. In addition, the result of duodenoscopy showed that Whipple surgery could be avoided because the duodenal papilla is not involved. It was gratified that the patient was very agreeable to our procedure plan.

**Figure 3:**
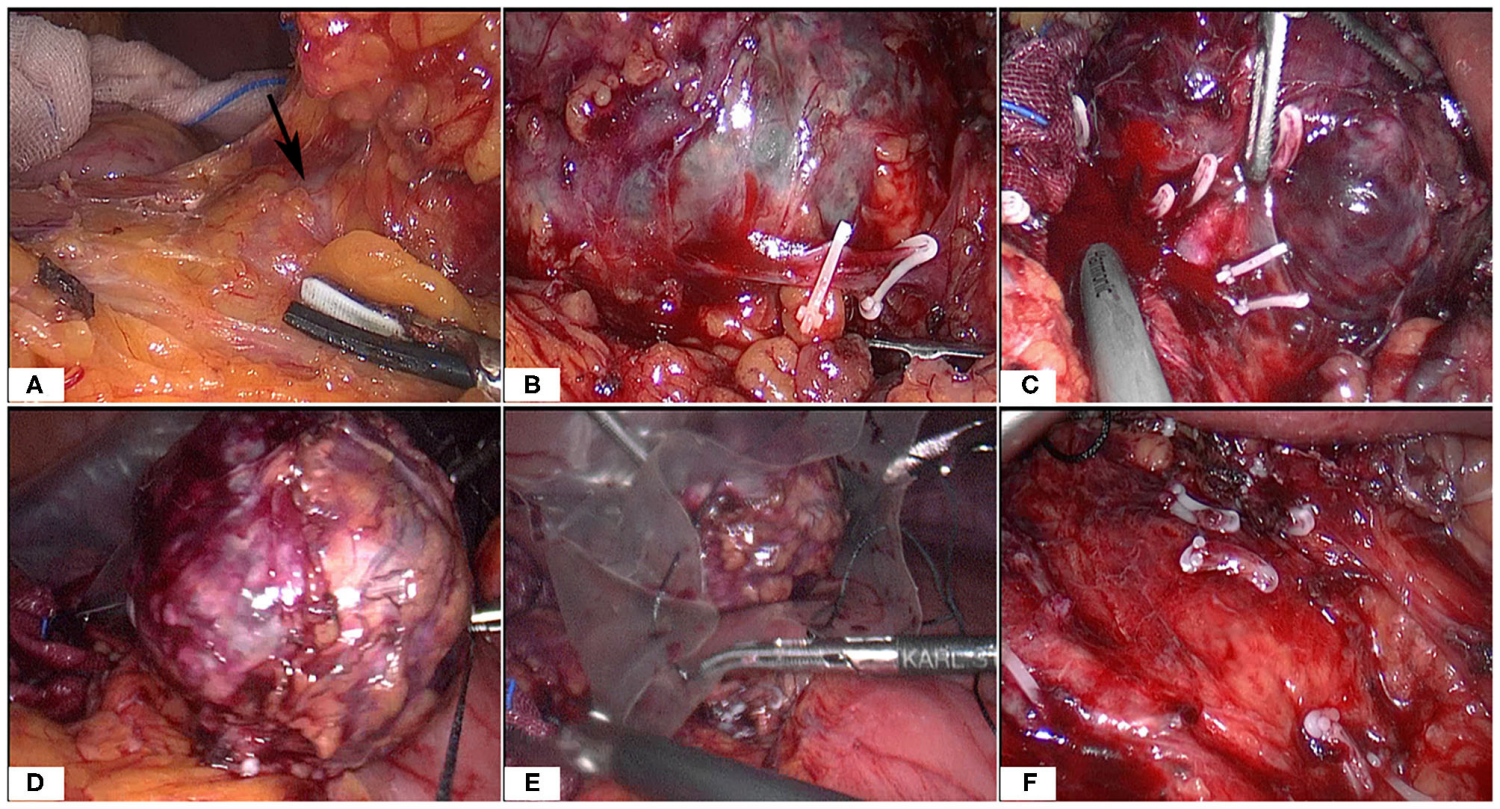
Intraoperative images **(A–F)**. Note the large tumor and multiple surrounding vessels which were clipped by Hem-o-Loks or ultrasonic Scalpel. Then the tumor was removed with an endobag.

**Figure 4:**
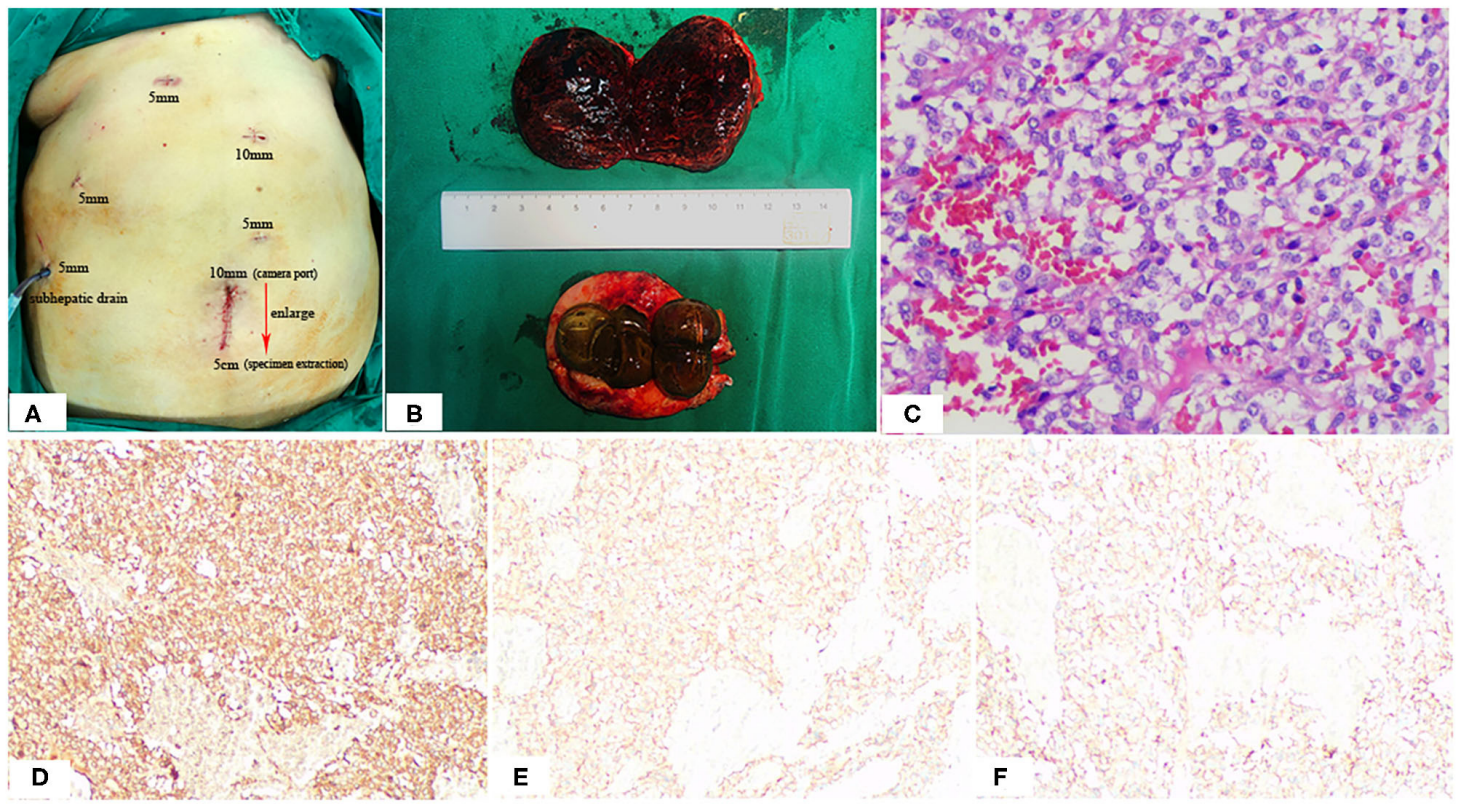
Positions of the ports **(A)**, Postoperative specimen **(B)**, and Pathologic Findings **(C)**. The 10-mm umbilical port (camera port) was enlarging to 5 cm to extract specimen **(A)**. Note the hemorrhage, cystic degeneration of the paraganglioma **(B)**. HE staining: **(C)** SP × 200; immunohistochemistry (IHC): **(D)** CgA (+), **(E)** Syn (+), **(F)** CD56 (+).

At surgery, the patient was positioned supine and the first 10 mm trocar was inserted below the umbilicus as a camera port. The trocar positioning, size and number depended on the localization of the tumor and surgeons' preference. In our case, because we need to perform a laparoscopic cholecystectomy for gallstones at the same time, We established six trocars, and other five additional trocars were placed under direct vision: two 5 mm trocars were placed on the right subcostal anterior axillary line and midclavicular line, and one 5 mm trocar was under the xiphisternum, subsequently, one 10 mm trocar and one 5 mm trocar were placed on the left subcostal anterior axillary line and midclavicular line (Figure 4A). The pressure of CO_2_ pneumoperitoneum was maintained at 12 mmHg. Intraoperative exploration found that the tumor was located in the retroperitoneum with no exposure into the abdominal cavity. And the tumor was showed to be retroperitoneal adherent to the 2th segment of the duodenum but without invasion. Thereafter careful dissection was performed to separate the tumor from the multiple surrounding feeding vessels and tissues using Hem-o-lok clips or Ultrasonic Scalpel. Finally, The mass was dissected free and which was placed in an endobag and removed by enlarging the 10-mm umbilical port (camera port) to 5 cm due to tumor size, and a subhepatic drain was placed (Figures 3, 4A). We did not encounter any intraoperative complications, and blood pressure, heart rate and other vital signs were stable throughout surgery (Figure 1B). The total operative time was 270 minutes (including laparoscopic cholecystectomy) and intraoperative blood loss was around 200 mL. After surgery, the patient underwent uneventful recovery and the subhepatic drain was removed on the 6th postoperative days, and the patient was discharged without complications on postoperative day 9.

## Pthologic Findings

The macroscopic appearance of the resected retroperitoneal specimen was a light red, soft, well-circumscribed mass, measured 6.4 × 5.4 cm, and had a fibrous capsule. In cross-section, the tumor was solid, dark red in color, partly mixed with hemorrhage and cystic degeneration. In addition, the gallbladder is filled with large stones (Figure 4B). Microscopically, the tumor cells are polygonal or oval with rich eosinophilic cytoplasm and no mitoses were observed. Immunohistochemically, the tumor cells were positive for chromogranin (CgA), synaptophysin (Syn) and CD56, but negative for CD34, CD117, Dog-1, S-100, CK, Vimentin and Calretinmin. And Ki-67 marker showed low mitotic index (Figures 4C–F). Lastly the diagnosis of retroperitoneal paraganglioma was confirmed based on these histopathological and immunohistochemical findings.

## Discussion

Paragangliomas are tumors derived from extra adrenal chromaffin tissue, which can be divided into parasympathetic and sympathetic types and the majority of RPGLs were sympathetic paragangliomas. Paragangliomas are classified as functioning and non-functioning tumors defined by the production of catecholamine (9). Due to the deep location of retroperitoneal paraganglioma, especially the non-functional tumor, and the lack of specific clinical manifestations and imaging manifestations, early diagnosis is difficult, and it is easy to cause missed diagnosis and misdiagnosis (4). As in our case, asymptomatic tumor disguised as subserosa type of duodenal GIST, which was not confirmed as paraganglioma until postoperative pathology.

Non-functional paragangliomas only show symptoms and signs caused by local occupation. In addition to local symptoms, functional paragangliomas also have a series of symptoms caused by excessive secretion of catecholamine, most of which are hypertension, headache, palpitation, hyperhidrosis and nausea (2, 10). Therefore, imaging examination is the most important diagnostic method, especially for non-functional RPGLs. Imaging studies allow assessment of the location, extent and multifocality of the disease (10). CT or MRI imaging features of paraganglioma are clear boundary, abundant blood supply, uneven texture, and some of them are accompanied with cystic or necrotic changes (11, 12). Although, CT and MRI both have high sensitivity to paraganglioma, they lack specificity (13). In CT or MRI imaging, RPGL and other retroperitoneal masses may have a similar imaging appearance, RPGL may be misdiagnosed as retroperitoneal tumors, such as submucosal tumor, retroperitoneal sarcoma, isolated adrenal hydatid cyst and duodenal GIST (like in our case) (2–5). Moreover, iodine 131 metaiodobenzylguanidine (MIBG) is also helpful in the diagnosis of RPGL, a structural analog of norepinephrine, which can achieve a diagnostic accuracy of more than 90%. And MIBG scintigraphy is less sensitive than CT and MRI but is highly specific for RPGL (13–15). MIBG can also distinguish functional from non-functional tumors and identify metastases (16). Besides, RPGL disease should also be considered in some patients who were not diagnosed preoperatively and had significantly increased blood pressure during intraoperative contact with the tumor. CT guided percutaneous biopsy is a reliable method for the diagnosis, and we should also pay attention to the potential risk of hypertension crisis. And, the diagnosis of malignant paragangliomas is based on evidence of extensive local invasion or metastatic disease rather than pathological diagnosis (17, 18).

Surgical resection is the preferred therapy for RPGL, however, it is still a great challenge for the operation of the tumor with abundant blood supply and complicated peripheral vascular growth. In the past, the treatment of paraganglioma was usually open surgery, but with the improvement of laparoscopic surgical skills and even the rise of robotic surgery, laparoscopic surgical resection has gradually become the preferred option for paraganglioma. Laparoscopic surgery has been successfully performed in patients with RPGL, and has achieved satisfactory results. For experienced surgeons, laparoscopic surgery remains an option even for patients with recurrent, metastatic tumors or giant sized neoplasms (≥6 cm) (7, 8, 19). The benefits of laparoscopic resection contrast to conventional open surgery include less postoperative pain, lower complication rates, less trauma and quick recovery. As in our case, the patient was very satisfied with the diagnosis and treatment of the whole perioperative period, who was underwent uneventful recovery and discharged without complications on postoperative day 9. In addition, in the laparoscopic procedure, magnified view and sufficient space provided by the laparoscope can theoretically improve operative precision (20). However, the laproscopic management is a more challenging technology and needs a longer learning curve. Laparoscopic surgery used for RPGLs includes transperitoneal and retroperitoneal approaches, and with the benefits of sufficient operation space and the easy exposure of tumor, transperitoneal approach is the main way of laparoscopic surgery. Surely, for transperitoneal approach, it is necessary to dissect adjacent structures for sufficient operation space and which consumes more surgical time. The advantages of the retroperitoneal approach include a shorter operation time and fewer disturbances of adjacent organs. As for retroperitoneal approach, it can reduce the risk of abdominal organ injury and shorten the operation time, but it has the disadvantages of having a smaller retroperitoneal operation space and fewer anatomical markers. We can choose different surgical approaches according to the relationship between the tumor and the large vessels such as renal vessels, aorta and vena cava. For the former, we choose retroperitoneoscopic approaches, while for the latter, tumors were removed using transperitoneoscopic approaches (6, 20). In addition, with the rise of robotic surgery by Leonardo Da Vinci, some medical centers have been using this method to break through the limit of tumor resection, which also has a good application prospect (21–23).

RPGL is a tumor with potential for biologically aggressive behavior and has a low risk of recurrence after the tumor removal. Previous studies have shown that tumor diameter > 6 cm, succinate dehydrogenase B mutation, non-functional tumor, Ki-67 index > 3% and with focal or mixed necrosis are the adverse factors affecting the prognosis of RPGL (24), and therefore lifelong annual follow-up in RPGL is recommended. So, annual clinical, laboratory test and imaging examination are essential in all RPGL patients after tumor resection. In case of local recurrence, surgery remains the primary choice.

In summary, RPGL is a rare tumor, and functional paragangliomas have a series of symptoms caused by excessive secretion of catecholamine while non-functional paragangliomas only show symptoms and signs caused by local occupation, which should be considered as a differential diagnosis of asymptomatic retroperitoneal tumor. RPGL is not sensitive to radiotherapy and chemotherapy thus surgical treatment is the first choice. In addition, the transperitoneal laparoscopic approach for the RPGL is a safe, applicable method with less trauma and quick recovery, which is worth clinical popularizing and application. Moreover, the survival prognosis of RPGL patients are related to metastasis, and lifelong follow-up should be emphasized.

## Data Availability Statement

The original contributions presented in the study are included in the article/supplementary material, further inquiries can be directed to the corresponding author/s.

## Ethics Statement

Written informed consent was obtained from the individual(s) for the publication of any potentially identifiable images or data included in this article.

## Author Contributions

ZZ was involved in the identification and selection of patient case and drafted the manuscript. ZT contributed to the conception of the manuscript. ZL and YL contributed to the patient care and management. ZZ, ZT, and JY reviewed and edited the manuscript. All authors read and approved the final manuscript.

## Conflict of Interest

The authors declare that the research was conducted in the absence of any commercial or financial relationships that could be construed as a potential conflict of interest.
